# 1,3,5-Tri­fluoro-2,4,6-tri­iodo­benzene–piperazine (2/1)

**DOI:** 10.1107/S2414314621010440

**Published:** 2021-10-13

**Authors:** Christelle Hajjar, Jeffrey S. Ovens, David L. Bryce

**Affiliations:** a Department of Chemistry and Biomolecular Sciences, University of Ottawa, Ottawa, ON K1N6N5, Canada; Howard University, USA

**Keywords:** crystal structure, halogen bonding, stoichiometry, co-crystal

## Abstract

The single-crystal structure of the title compound, C_4_H_10_N_2_·2C_6_F_3_I_3_, features a moderately strong halogen bond between one of the three crystallographically distinct iodine atoms and the nitro­gen atom. The iodine–nitro­gen distance is 2.820 (3) Å, corresponding to 80% of the sum of their van der Waals radii. The C—I⋯N halogen bond angle is 178.0 (1)°, consistent with the linear inter­action of nitro­gen *via* a σ-hole opposite the carbon–iodine covalent bond. The other two iodine atoms do not engage in halogen bonding. Some weak C—H⋯F and —H⋯I interactions are also observed. The complete piperazine molecule is generated by symmetry.

## Abstract 2

The single-crystal structure of the title compound features a moderately strong halogen bond between one of the three crystallographically distinct iodine atoms and the nitro­gen atom. The other two chemically identical iodine atoms do not engage in halogen bonding.

## Graphical abstract

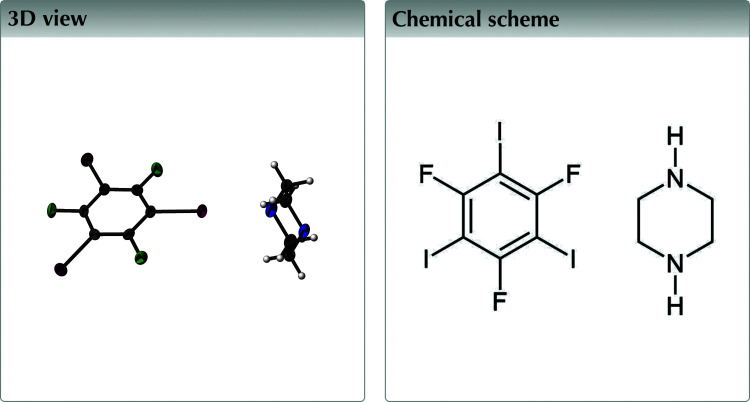

## Structure description

**Figure 1:**
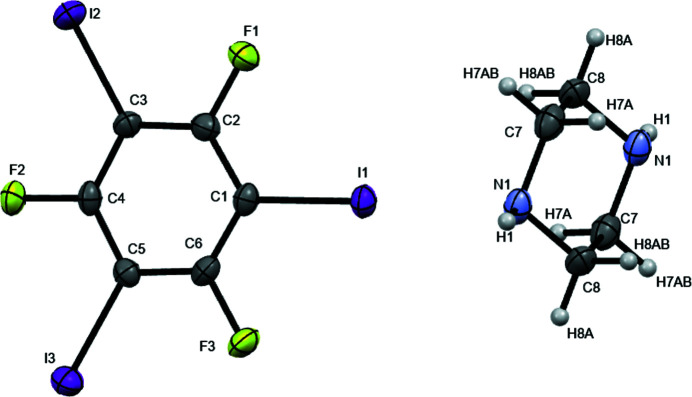
*ORTEP* plot of the title compound.

**Figure 2:**
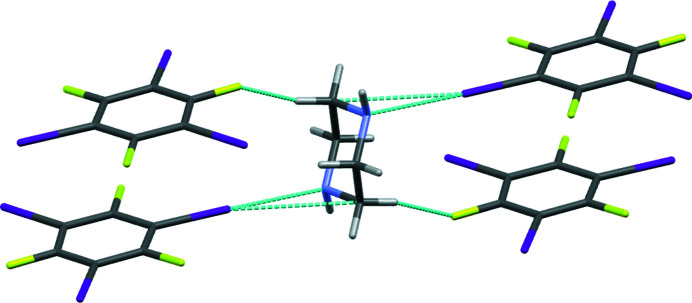
Detail of the X-ray crystal structure depicting a halogen bond between iodine and nitro­gen, a short contact between iodine and carbon, and a short contact between hydrogen and fluorine. The other two iodine atoms on the aromatic ring do not engage in any halogen bonding or other close contacts.

The halogen bond is a moderately strong and directional non-covalent inter­action, which has proven very useful in the field of crystal engineering and for the design of co-crystalline materials. Perfluorinated iodo­benzenes are commonly used as halogen-bond donors, in part due to their reliable ability to co-crystallize predictably with a range of electron donors (Cavallo, 2016 ▸). The title compound (Fig. 1 ▸), which has a 2:1 1,3,5-tri­fluoro-2,4,6-tri­iodo­benzene:piperazine (1,4-di­aza­cyclo­hexa­ne) stoichiometry, features a halogen bond between I1 as the halogen-bond donor and N1 as the halogen-bond acceptor (Fig. 2 ▸). The iodine–nitro­gen distance is 2.820 (3) Å, which corresponds to 80% of the sum of their van der Waals radii. This is somewhat shorter than the analogous iodine–nitro­gen halogen bonds in co-crystals formed from the same halogen-bond donor with acridine (3.022 Å), 1,10-phenanthroline (3.020 and 3.148 Å), or 2,3,5,6-tetra­methyl­pyrazine (2.991 and 2.993 Å), but comparable to those formed with hexa­methyl­ene­tetra­mine (2.864 and 2.879 Å) as the electron donor (Szell *et al.*, 2017 ▸). Comparable distances are also noted in an inter­esting class of halogen-bonded tubular structures formed from the self-assembly of 1,4-di­iodo­tetra­fluoro­benzene and piperazine cyclo­phanes (Raatikainen, 2009 ▸).

**Figure 3:**
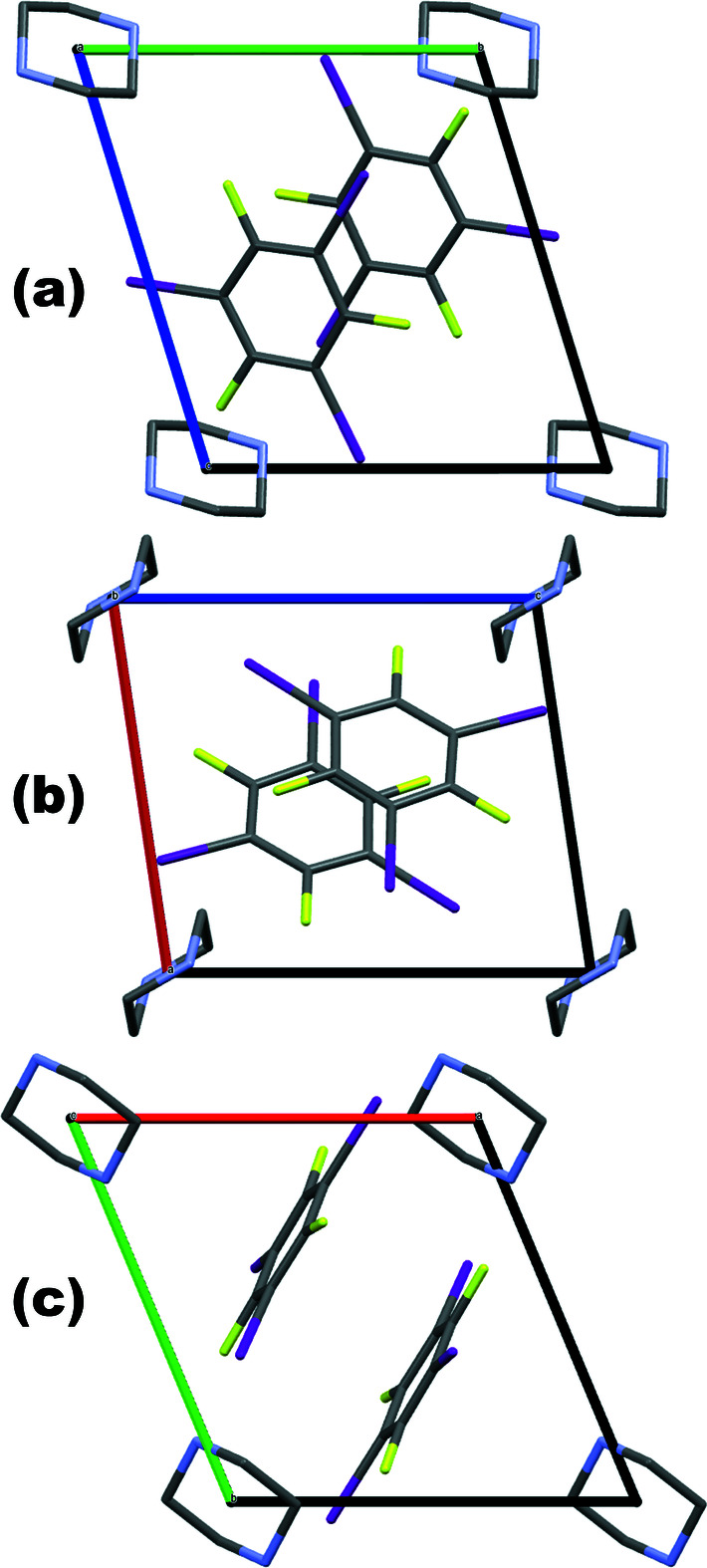
View along each of the unit cell axes. (*a*): along the *a* axis; (*b*): along the *b* axis; (*c*): along the *c* axis. Hydrogen atoms not shown.

The C1—I1⋯N1 halogen bond angle in the title compound is 178.0 (1)°, consistent with the linear inter­action of nitro­gen *via* a σ-hole opposite the carbon–iodine covalent bond. I1 also shows a short contact with C7 of the piperazine mol­ecule of 3.578 (4) Å; this represents approximately 97% of the sum of their van der Waals radii and is likely a structural consequence of the formation of the adjacent halogen bond rather than a structure-directing element in and of itself. Possible weak hydrogen bonds are also observed between H1 and I2, between H7*A* and F2, between H7*AB* and I3, between H8*A* and I2, and between H8*AB* and I1 (Table 1 ▸). Inter­estingly, no halogen bonds involving I2 and I3 are observed, despite the fact that they are chemically identical to I1. The structure packs in the triclinic *P* 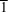 space group and the aromatic mol­ecules lie in layers (Fig. 3 ▸). The stoichiometry of the co-crystal is highlighted by noting that pairs of aromatic mol­ecules lying in adjacent layers are connected to each other *via* halogen bonding to a single common piperazine mol­ecule.

## Synthesis and crystallization

1,3,5-Tri­fluoro-2,4,6-tri­iodo­benzene was purchased from Alfa Aesar and piperazine was purchased from Sigma–Aldrich. In a typical procedure, the title compound was obtained from the slow evaporation of a solution of the halogen-bond donor (0.025 g in 1 ml of chloro­form) and a molar excess of halogen-bond acceptor (0.0412 g in 1 ml of ethanol) at room temperature. The two solutions were prepared independently and stirred. After dissolution, the two solutions were mixed, stirred, and covered to allow for slow evaporation and crystal formation.

## Refinement

Crystal data, data collection, and structure refinement details are presented in Table 2 ▸.

## Supplementary Material

Crystal structure: contains datablock(s) I. DOI: 10.1107/S2414314621010440/bv4042sup1.cif

Structure factors: contains datablock(s) I. DOI: 10.1107/S2414314621010440/bv4042Isup2.hkl

Supporting information file. DOI: 10.1107/S2414314621010440/bv4042Isup3.cml

CCDC reference: 2101749

Additional supporting information:  crystallographic information; 3D view; checkCIF report

## Figures and Tables

**Table 1:** Hydrogen-bond geometry (Å, °)

*D*—H⋯*A*	*D*—H	H⋯*A*	*D*⋯*A*	*D*—H⋯*A*
N1—H1⋯I2^i^	0.86 (2)	3.12 (2)	3.978 (3)	177 (3)
C7—H7*A*⋯F2^ii^	0.98	2.40	3.299 (4)	152
C7—H7*AB*⋯I3^iii^	0.98	3.23	3.990 (3)	135
C8—H8*A*⋯I2^iv^	0.98	3.19	4.001 (3)	141
C8—H8*AB*⋯I1^v^	0.98	3.26	3.879 (3)	123

Symmetry codes: (i) 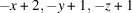 ; (ii) 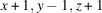 ; (iii) 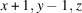 ; (iv) 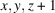 ; (v) 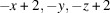 .

**Table 2:** Experimental details

Crystal data	
Chemical formula	C_4_H_10_N_2_·2C_6_F_3_I_3_
*M*_r_	1105.66
Crystal system, space group	Triclinic, *P* 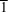
Temperature (K)	203
*a*, *b*, *c* (Å)	8.6450 (5), 9.1660 (5), 9.3403 (5)
α, β, γ (°)	67.433 (1), 72.887 (1), 63.062 (1)
*V* (Å^3^)	602.75 (6)
*Z*	1
Radiation type	Mo *K*α
μ (mm^−1^)	7.78
Crystal size (mm)	0.24 × 0.13 × 0.08

Data collection	
Diffractometer	Bruker APEXII CCD
Absorption correction	Multi-scan (*SADABS*; Krause et al., 2015 ▸)
*T*_min_, *T*_max_	0.537, 0.746
No. of measured, independent and observed [*I* > 2σ(*I*)] reflections	13748, 3761, 3193
*R*_int_	0.031
(sin θ/λ)_max_ (Å^−1^)	0.721

Refinement	
*R*[*F* ^2^ > 2σ(*F* ^2^)], *wR*(*F* ^2^), *S*	0.025, 0.048, 1.02
No. of reflections	3761
No. of parameters	139
No. of restraints	1
H-atom treatment	H atoms treated by a mixture of independent and constrained refinement
Δρ_max_, Δρ_min_ (e Å^−3^)	0.56, −0.64

Computer programs: *APEX3* and *SAINT* (Bruker, 2010 ▸), *SHELXT2014/5* (Sheldrick, 2015*a* ▸), *SHELXL2018*/3 (Sheldrick, 2015*b* ▸), *ShelXle* (Hübschle *et al.*, 2011 ▸) and *Mercury* (Macrae *et al.*, 2020 ▸).

## full crystallographic data

### Crystal data

**Table:**

C_4_H_10_N_2_·2C_6_F_3_I_3_	*Z* = 1
*M**_r_* = 1105.66	*F*(000) = 492
Triclinic, *P*1	*D*_x_ = 3.046 Mg m^−^^3^
*a* = 8.6450 (5) Å	Mo *K*α radiation, λ = 0.71073 Å
*b* = 9.1660 (5) Å	Cell parameters from 6804 reflections
*c* = 9.3403 (5) Å	θ = 2.4–30.7°
α = 67.433 (1)°	µ = 7.78 mm^−^^1^
β = 72.887 (1)°	*T* = 203 K
γ = 63.062 (1)°	Block, colourless
*V* = 602.75 (6) Å^3^	0.24 × 0.13 × 0.08 mm

### Data collection

**Table:**

Bruker APEXII CCD diffractometer	3193 reflections with *I* > 2σ(*I*)
Graphite monochromator	*R*_int_ = 0.031
ω and πhi scans	θ_max_ = 30.8°, θ_min_ = 2.4°
Absorption correction: multi-scan (SADABS; Krause et al., 2015\bbr00)	*h* = −12→12
*T*_min_ = 0.537, *T*_max_ = 0.746	*k* = −13→13
13748 measured reflections	*l* = −13→13
3761 independent reflections	

### Refinement

**Table:**

Refinement on *F*^2^	Primary atom site location: structure-invariant direct methods
Least-squares matrix: full	Hydrogen site location: mixed
*R*[*F*^2^ > 2σ(*F*^2^)] = 0.025	H atoms treated by a mixture of independent and constrained refinement
*wR*(*F*^2^) = 0.048	*w* = 1/[σ^2^(*F*_o_^2^) + (0.0176*P*)^2^ + 0.1043*P*] where *P* = (*F*_o_^2^ + 2*F*_c_^2^)/3
*S* = 1.02	(Δ/σ)_max_ = 0.001
3761 reflections	Δρ_max_ = 0.56 e Å^−^^3^
139 parameters	Δρ_min_ = −0.64 e Å^−^^3^
1 restraint	

### Special details

**Table:**

Experimental. Crystallographic data were collected from single crystals mounted on MiTeGen MicroMounts using parabar oil. Data were collected on a Bruker SMART APEXII single-crystal diffractometer equipped with a sealed tube Mo Kα source (λ= 0.71073 Å), a graphite monochromator, and an APEXII CCD detector. Samples were held at low temperature using a dry compressed air cooling system. Raw data collection and processing were performed with the *APEX3* software package from Bruker (2010). Initial unit-cell parameters were determined from 36 data frames from select ω scans. Semi-empirical absorption corrections based on equivalent reflections were applied (Blessing, 1995). Systematic absences in the diffraction data-set and unit-cell parameters were consistent with the assigned space group. The initial structural solutions were determined using *SHELXT* direct methods (Sheldrick, 2015*a*) and refined with full-matrix least-squares procedures based on *F*_2_ using *SHELXL* and *ShelXle* (Hübschle *et al.*, 2011; Sheldrick, 2015*b*). Hydrogen atoms were placed geometrically and refined using a riding model.
Geometry. All esds (except the esd in the dihedral angle between two l.s. planes) are estimated using the full covariance matrix. The cell esds are taken into account individually in the estimation of esds in distances, angles and torsion angles; correlations between esds in cell parameters are only used when they are defined by crystal symmetry. An approximate (isotropic) treatment of cell esds is used for estimating esds involving l.s. planes.
Refinement. none

### Fractional atomic coordinates and isotropic or equivalent isotropic displacement parameters (Å^2^)

**Table:**

	*x*	*y*	*z*	*U*_iso_*/*U*_eq_	
I1	0.82683 (3)	0.38276 (3)	0.69997 (2)	0.02587 (5)	
N1	1.0231 (4)	0.1572 (3)	0.9473 (3)	0.0297 (6)	
H1	1.079 (4)	0.212 (4)	0.952 (4)	0.036*	
C1	0.6818 (4)	0.5589 (4)	0.5141 (3)	0.0236 (6)	
F1	0.8619 (2)	0.3929 (2)	0.3438 (2)	0.0350 (5)	
I2	0.70010 (3)	0.60960 (3)	0.02390 (2)	0.03224 (6)	
C2	0.7265 (4)	0.5356 (4)	0.3663 (4)	0.0246 (6)	
F2	0.4127 (2)	0.9155 (2)	0.1493 (2)	0.0305 (4)	
I3	0.22867 (3)	1.04531 (3)	0.44595 (3)	0.03033 (6)	
C3	0.6376 (4)	0.6522 (4)	0.2419 (3)	0.0226 (6)	
F3	0.4867 (2)	0.7292 (2)	0.6764 (2)	0.0334 (4)	
C7	1.1580 (4)	0.0068 (4)	0.8988 (4)	0.0316 (7)	
H7A	1.243223	−0.060464	0.972668	0.038*	
H7AB	1.220520	0.044203	0.794519	0.038*	
C6	0.5381 (4)	0.7041 (4)	0.5336 (3)	0.0235 (6)	
C5	0.4444 (4)	0.8275 (4)	0.4131 (3)	0.0218 (6)	
C4	0.4991 (4)	0.7972 (4)	0.2689 (3)	0.0225 (6)	
C8	0.9301 (4)	0.1016 (4)	1.1043 (4)	0.0315 (7)	
H8A	0.841947	0.201779	1.136757	0.038*	
H8AB	1.013321	0.034933	1.180012	0.038*	

### Atomic displacement parameters (Å^2^)

**Table:**

	*U*^11^	*U*^22^	*U*^33^	*U*^12^	*U*^13^	*U*^23^
I1	0.02951 (11)	0.02437 (10)	0.02130 (10)	−0.00916 (8)	−0.00948 (8)	−0.00148 (8)
N1	0.0415 (17)	0.0289 (15)	0.0227 (14)	−0.0176 (13)	−0.0112 (12)	−0.0020 (12)
C1	0.0253 (15)	0.0261 (16)	0.0199 (15)	−0.0109 (12)	−0.0078 (12)	−0.0028 (12)
F1	0.0367 (11)	0.0271 (10)	0.0300 (11)	0.0021 (8)	−0.0089 (8)	−0.0115 (8)
I2	0.04118 (13)	0.03267 (12)	0.02151 (11)	−0.01026 (9)	−0.00498 (9)	−0.01114 (9)
C2	0.0241 (15)	0.0223 (15)	0.0255 (16)	−0.0074 (12)	−0.0017 (12)	−0.0082 (13)
F2	0.0380 (11)	0.0267 (10)	0.0211 (9)	−0.0063 (8)	−0.0141 (8)	−0.0016 (8)
I3	0.02595 (11)	0.02909 (11)	0.02966 (12)	−0.00356 (8)	−0.00403 (8)	−0.01089 (9)
C3	0.0276 (15)	0.0227 (15)	0.0191 (15)	−0.0091 (12)	−0.0049 (12)	−0.0074 (12)
F3	0.0358 (11)	0.0399 (11)	0.0175 (9)	−0.0072 (9)	−0.0039 (8)	−0.0104 (8)
C7	0.0280 (17)	0.0381 (19)	0.0249 (17)	−0.0119 (14)	−0.0076 (13)	−0.0036 (14)
C6	0.0272 (16)	0.0287 (16)	0.0162 (14)	−0.0137 (13)	−0.0017 (11)	−0.0058 (12)
C5	0.0203 (14)	0.0207 (14)	0.0239 (15)	−0.0073 (11)	−0.0039 (11)	−0.0061 (12)
C4	0.0242 (15)	0.0221 (15)	0.0216 (15)	−0.0106 (12)	−0.0103 (12)	0.0002 (12)
C8	0.042 (2)	0.0293 (18)	0.0219 (16)	−0.0107 (15)	−0.0069 (14)	−0.0092 (14)

### Geometric parameters (Å, º)

**Table:**

I1—C1	2.118 (3)	I3—C5	2.078 (3)
N1—C8	1.470 (4)	C3—C4	1.379 (4)
N1—C7	1.475 (4)	F3—C6	1.350 (3)
N1—H1	0.864 (18)	C7—C8^i^	1.513 (5)
C1—C6	1.380 (4)	C7—H7A	0.9800
C1—C2	1.392 (4)	C7—H7AB	0.9800
F1—C2	1.343 (3)	C6—C5	1.390 (4)
I2—C3	2.089 (3)	C5—C4	1.381 (4)
C2—C3	1.382 (4)	C8—H8A	0.9800
F2—C4	1.345 (3)	C8—H8AB	0.9800
			
C8—N1—C7	110.0 (2)	H7A—C7—H7AB	108.3
C8—N1—H1	108 (2)	F3—C6—C1	118.5 (3)
C7—N1—H1	106 (2)	F3—C6—C5	118.1 (3)
C6—C1—C2	116.5 (3)	C1—C6—C5	123.3 (3)
C6—C1—I1	121.3 (2)	C4—C5—C6	116.7 (3)
C2—C1—I1	122.2 (2)	C4—C5—I3	120.8 (2)
F1—C2—C3	118.5 (3)	C6—C5—I3	122.5 (2)
F1—C2—C1	118.4 (3)	F2—C4—C3	118.6 (3)
C3—C2—C1	123.0 (3)	F2—C4—C5	118.1 (3)
C4—C3—C2	117.1 (3)	C3—C4—C5	123.3 (3)
C4—C3—I2	120.5 (2)	N1—C8—C7^i^	109.4 (3)
C2—C3—I2	122.4 (2)	N1—C8—H8A	109.8
N1—C7—C8^i^	109.0 (3)	C7^i^—C8—H8A	109.8
N1—C7—H7A	109.9	N1—C8—H8AB	109.8
C8^i^—C7—H7A	109.9	C7^i^—C8—H8AB	109.8
N1—C7—H7AB	109.9	H8A—C8—H8AB	108.2
C8^i^—C7—H7AB	109.9		
			
C6—C1—C2—F1	−177.8 (3)	F3—C6—C5—C4	−179.3 (3)
I1—C1—C2—F1	2.8 (4)	C1—C6—C5—C4	1.5 (5)
C6—C1—C2—C3	2.0 (5)	F3—C6—C5—I3	−0.7 (4)
I1—C1—C2—C3	−177.5 (2)	C1—C6—C5—I3	−179.9 (2)
F1—C2—C3—C4	179.9 (3)	C2—C3—C4—F2	178.3 (3)
C1—C2—C3—C4	0.1 (5)	I2—C3—C4—F2	−4.3 (4)
F1—C2—C3—I2	2.5 (4)	C2—C3—C4—C5	−1.6 (5)
C1—C2—C3—I2	−177.3 (2)	I2—C3—C4—C5	175.8 (2)
C8—N1—C7—C8^i^	60.1 (3)	C6—C5—C4—F2	−179.0 (3)
C2—C1—C6—F3	177.9 (3)	I3—C5—C4—F2	2.4 (4)
I1—C1—C6—F3	−2.6 (4)	C6—C5—C4—C3	0.8 (4)
C2—C1—C6—C5	−2.8 (5)	I3—C5—C4—C3	−177.8 (2)
I1—C1—C6—C5	176.6 (2)	C7—N1—C8—C7^i^	−60.3 (4)

Symmetry code: (i) −*x*+2, −*y*, −*z*+2.

### Hydrogen-bond geometry (Å, º)

**Table:**

*D*—H···*A*	*D*—H	H···*A*	*D*···*A*	*D*—H···*A*
N1—H1···I2^ii^	0.86 (2)	3.12 (2)	3.978 (3)	177 (3)
C7—H7*A*···F2^iii^	0.98	2.40	3.299 (4)	152
C7—H7*AB*···I3^iv^	0.98	3.23	3.990 (3)	135
C8—H8*A*···I2^v^	0.98	3.19	4.001 (3)	141
C8—H8*AB*···I1^i^	0.98	3.26	3.879 (3)	123

Symmetry codes: (i) −*x*+2, −*y*, −*z*+2; (ii) −*x*+2, −*y*+1, −*z*+1; (iii) *x*+1, *y*−1, *z*+1; (iv) *x*+1, *y*−1, *z*; (v) *x*, *y*, *z*+1.
